# A method for intracerebroventricular cannulation of young broiler chicks

**DOI:** 10.1016/j.mex.2022.101724

**Published:** 2022-05-05

**Authors:** Chris Lamberigts, Zhigang Song, Johan Buyse

**Affiliations:** a Laboratory of Livestock Physiology, Department of Biosystems, KU Leuven, Kasteelpark Arenberg 30, Leuven 3001, Belgium; b Department of Animal Science, Shandong Agricultural University, 61 Daizong Street, Taian, China

**Keywords:** Stereotaxic, ICV, Hypothalamus, Third ventricle, Broiler chicks, i.c.v, intracerebroventricular, aCSF, artificial cerebrospinal fluid

## Abstract

The site-specific administration of neuroactive substances or pharmacological agents is a routine practice in neurological studies. To facilitate chronic treatments intercranial cannulae are often implanted in the skull. These surgical procedures are widely described and well-documented for rodents, as the most common animal model, and even refined over the years. However, precise descriptions of proper procedure for third ventricle cannulation in young broiler chicks is not readily available. This absence is surprising, as chicken models are often used in the investigation of physiological control of the metabolism and ingestion. Furthermore, as a commercial animal, there is also a particular interest in elucidating the central regulation of feed intake and metabolism to optimize feeding and living conditions for broilers. The brain nuclei that serve as the regulatory centers of feeding and metabolism are the arcuate nucleus, the ventromedial hypothalamic nucleus, and the lateral hypothalamus, which are all located near the third ventricle. Here, we provide a full protocol for intracerebroventricular (i.c.v.) cannulation of 7-day old broiler chicks, as well as a review of some of the advances made in rodent i.c.v. cannulations and whether these advances are applicable to cannulations in chickens. Using the surgical procedure described here, we were able to achieve a success rate of proper implantation of 88%, with a mortality rate of less than 1% (*n* = 224).

• Detailed procedure for i.c.v. canula implantation in the third ventricle of 7d-old broiler chicks.
• Cement cap with anchoring screws is necessary in broilers, glue does not provide sufficient stability.
• Carboxylate cement and self-adhesive resin cement were tested as an alternative for methyl methacrylate cement.

## Graphical abstract

**Figure:**
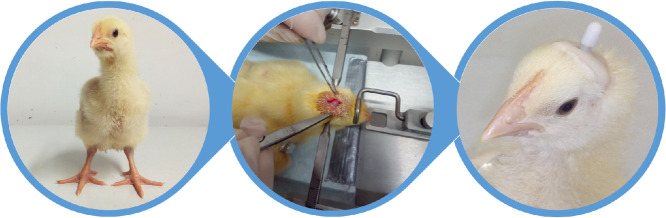

Specifications table

**Table:** 

Subject Area:	
More specific subject area:	*Neuroscience in Poultry*
Method name:	*Stereotaxic Implantation of an* ICV *Cannula in Young Broilers*
Name and reference of original method:	*N.A.*
Resource availability:	*N.A.*

## Method details

### Required equipment

- Stereotaxic apparatus: we used a ZH-lanxingC (Huaibei Zenghua Ltd. Co, Anhui, China), but any stereotaxic apparatus designed for rodents should do for chicks this age. A standard rat adapter proved ideal to gently but firmly fixate the chick's beak.
- Single guide cannulas (26-gauge, 9 mm in length beneath the pedestal)
- Dummy cannulas (30-gauge, 9 mm in length beneath the pedestal)
- Single injector (30-gauge, tip extending 0.5 mm beyond the guide cannula)
- 1,2 mm × 2 mm stainless steel screws
- Dental cement: dental resin and methyl methacrylate
- Dental drill with a foot pedal
- 1 mm drill bit
- Electric heating pad
- Ethanol
- Disinfectant wipes
- Needles
- Syringes
- 10 µl glass, air-tight Hamilton syringes with needle gauge 29 (1 per experimental group or per substance you want to inject)
- PTFE tubing, sterile (inner diameter 0.3 mm)
- Underpads
- Sterile gloves
- Tweezers
- Scalpel
- Scissors, both with straight and with curved blades
- Zoletil, or other appropriate analgesics/anesthetics
- Q-tips (sterile)

### Preparations

This procedure was optimized to be carried out in young broiler chicks (postnatal day 7-8), weighing approximately 90 to 120 g.

For injections, artificial cerebrospinal fluid (aCSF) was used as vehicle solution: 119 mM NaCl (Carl Roth GmBh, Karlsruhe, Germany), 25.2 mM NaHCO_3_ (Sigma-Aldrich, Saint Louis, MO, USA), 2.5 mM KCl (Honeywell Riedel-de-Haën AG, Seelze, Germany), 1 mM NaH_2_PO_4_ (Merck KGaA, Darmstadt, Germany), 1.3 MgCl_2_ (Merck KGaA) and 10 mM glucose (Sigma-Aldrich). Prepare and sterilize aCSF with 0.1% Evans Blue dye (Sigma-Aldrich). The dye is added to facilitate the localization of injection site.

Further preparations:

- Abstain the chicks from feed 4 h prior to anesthesia
- Set up the stereotaxic instrument to ensure the animal is positioned in the center of the frame
- Disinfect your working area
- Organize and sterilize all surgical equipment, instruments, and disposables
- Heat up sterile physiological saline to 42 °C
- Turn on the heating pad and stabilize the temperature at 40-42 °C

### Procedure

1. Sedation with Zoletil (consists of tiletamine and zolazepam; Virbac, Carros, France)
  a. Weigh the chick to determine the correct dose
  b. Intramuscular injection of 6.5 mg/kg body weight Zoletil [1]
  c. While the sedation sets in, clip the feathers on the head with curved-bladed mayo scissors
  d. Confirm the effect of sedation with a toe and/or comb pinch
2. Position the chick in a prone position on an underpad on top of a heating pad and fix the head into the stereotaxic frame
  a. Carefully insert the tip of the left ear bar into the left auditory canal. Take care not to insert the bar too deep to avoid damaging the tympanic membrane; it is sufficient if the skull is being carried by the bar.
  b. Next, carefully insert the tip of the right ear bar into the right auditory canal
  c. Center the chick's head in the stereotaxic frame and fix the ear bars in place
  d. Fixate the chick's beak in the clamp and adjust its height until the skull surface is horizontally positioned and the zygomatic bone is in line with the ear bars
  e. Clean and disinfect the chick's head with alcoholic swabs (70% ethanol)
  f. Change your gloves to a fresh sterile pair and cover the animal's body with a surgical field
3. Apply a local anesthetic, e.g. a topical 2% xylocaine gel, and make an incision in the midline of the scalp by gently lifting the skin with sterile tweezers and using either scissors or a scalpel blade. Clip the incision open to expose the skull
4. Use a sterile Q-tip to clear away blood and connective tissue until the skull is dry and bregma is clearly visible
5. Gently scratch the surface of the skull with a sterile surgical blade in a checkered pattern to provide additional adhesion for the cement cap
6. Using a dental drill with a 1 mm drill bit, drill 3 holes in the skull in a triangular array around the cannula insertion place. Apply warm, sterile physiological saline solution to reduce the heat during drilling.
  a. The screws will serve as an anchoring point for the cannula
  b. Screw holes should be as deep as possible without penetrating the skull completely
  c. Use sterile Q-tips to clear away the saline solution and blood, if any
7. Screw in the screws using tweezers and an appropriate screwdriver, taking care not to touch or irritate the dura. Make sure the screws are not flush up against the skull
8. Place a guide cannula with the dummy cannula inserted in the stereotaxic arm and position it directly above bregma
9. Move the guide cannula 2.0 mm anterior and 0.3 mm lateral from bregma
10. Pierce the dura at the insertion site with a sterile 21-gauge needle
11. Slowly lower and insert the guide cannula
12. Prepare the dental cement: mix acrylic cement powder with methyl methacrylate in a 1:1 ratio
13. Cover as much of the skull as you can with the skin flaps without covering the screws
14. Fix the entire assembly in place: apply the dental cement and make sure it fills the area under the screw heads
15. When the cement is (almost) dry, carefully release the guide cannula from the stereotaxic arm
16. Release the beak clamp, remove the ear bars, and allow the animal to recover for at least 5 days before the actual injections starts

### Manual injections through the cannula

1. During the recovery period, handle the chicks daily and make sure to unscrew and re-screw the dummy cannula at least once a day. This should familiarize the animals with the injection procedure, limits their stress levels and allows you to check on the stability of the guide cannula.
2. Connect an injector cannula to a 10 µl Hamilton microsyringe through PTFE tubing
3. Fill the tube partly with the desired drug for injection
4. Unscrew the dummy cannula, insert the injector cannula, and slowly deliver the drug over a period of 1 min
5. Leave the injector cannula in place for an additional minute to prevent backflow of fluids
6. Replace the injector cannula with the dummy cannula

## Rationale, tricks and tips

Take note that this procedure cannot be used if you want to assess performance parameters in broiler chicks. In our experience, chicks will lag behind on commercial performance targets due to the fasting prior to the surgery and the recovery time afterwards, although Shiraishi et al. [2] reported that when the cannula is implanted shortly after hatching there was no difference in body weight gain, feed intake or heart, liver, pancreas, and pectoral muscle weight between control chicks and 11d-old cannulated chicks.

**Fig. 1:**
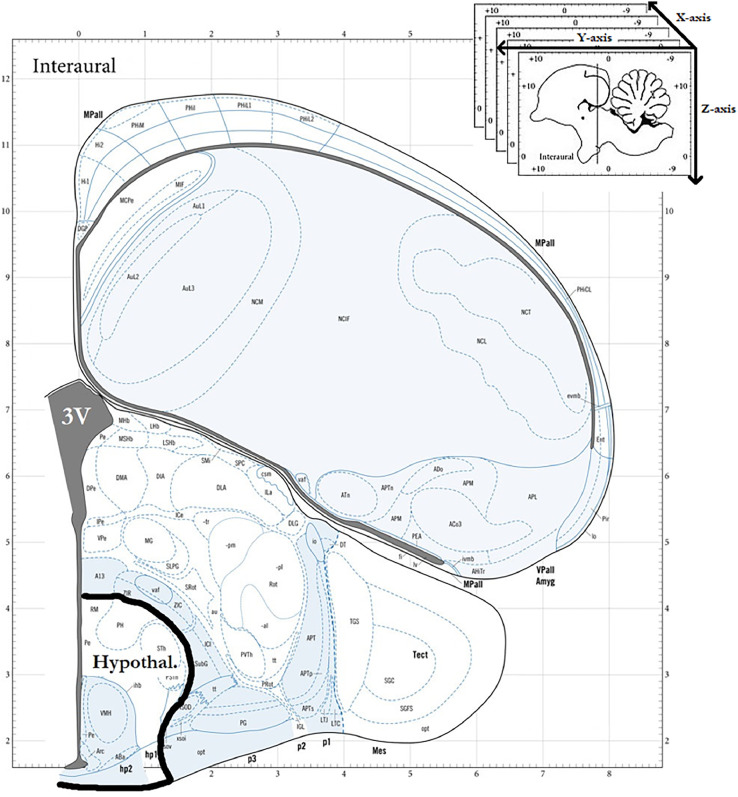
Schematic of the coronal section used for determining the placement of the cannula into the arcuate hypothalamic nucleus. Reference axes X/Y/Z are mediolateral/ anterior-posterior/ vertical. 3V = the third ventricle; Hypothal. = hypothalamus. Image based on “The Chick Brain in Stereotaxic Coordinates” Puelles et al., 2019.

The coordinates for the implantation site were determined using the Chick Brain Atlas [3] as depicted in Fig. 1: 2.0 mm anterior from bregma and 0.3 mm lateral. The lateral coordinates are automatically achieved if the guide cannula is properly centered along the midline. Most brain atlases use the interaural line as reference point, but bregma is more commonly used now (the intersection of the sagittal suture with the coronal suture). Preference for bregma stems from its high visibility and the possibility of keeping the head wound as minimal as possible. At this age, the sagittal suture is still soft and the cannula can be placed without drilling a hole at the insertion site.

While optimizing this protocol several approaches were tested to stabilize the cannula. These days, when performing cannula implantations in rodents, it is common to fixate the cannula with cyanoacrylic glue without the use of anchoring screws [4]. This approach does not only decrease the time spent in surgery, it has the additional advantage of resulting in a much smaller incision site and postoperative wound, speeding up the recovery of the animals tremendously. In our approach, we have tested Loctite 454 and Loctite Superglue 3 (Henkel, Düsseldorf, Germany) for this purpose [5]. Initially, using cyanoacrylic glue yielded very satisfying results. Loctite Superglue 3 dried faster than Loctite 454, but had a shiny finish. For chicks, using the Loctite 454 with a more matte finish would be better, as chicks tend to peck at shiny objects. However, due to chicken's pecking behavior in general, it turned out that the cannula could not be stabilized enough without the use of anchoring screws. Chickens spend most of their active time pecking and preening, sometimes bumping their head on obstacles in the process. Many cannulas quickly dislodged during feeding, and the use of cyanoacrylic glue for i.c.v. cannulation in chicks was abandoned.

Two alternatives to methyl methacrylate cement were explored. The use of methyl methacrylate dental cement to stabilize head attachments is well-established in laboratory animals but has 2 major downfalls: the bonding capacity to bioactive surfaces [6] such as the skull is relatively low and the polymerization process is an exothermic reaction. Temperatures as high as 81,3 °C were registered - measured with an infrared thermometer (Raynger PM4; Raytek, Santa Cruz, CA, USA) - resulting in necrotic tissue underneath the methyl methacrylate cap. When using carboxylate cement as an alternative, Poly-F Plus (Dentsply Sirona, York, PA, USA), it too resulted in necrotic tissue contrary to the report of Agterberg et al. [5] and did not provide the necessary stability. It seemed like water was expelled during the polymerization reaction of the cement, hampering bonding to the skull and overall stability of the cement cap. A second alternative was a self-adhesive resin cement, RelyX Unicem 2 (3M, Maplewood, Minnesota, USA), which was provided in a pistol with double plunger, mixing 2 components upon application. This cement proved adequate but is costly and a substantial amount is needed to form the caps.

As an alternative to scratching the skull with a surgical blade to increase the bonding of the cement to the skull, the skull can be etched with 1 mmol/L HCl and subsequently rinsed with sterile saline. However, this chemical method is slightly more labor intensive and does not provide a better bonding strength compared to scratching.

## Method validation

### Angiotensin check

When applied directly to the brain, angiotensin II triggers a strong urge to drink. The anterior hypothalamus is the brain's most sensitive area to angiotensin [7] and the dipsogenic response is copious and rapid. Each chick that did not start drinking within 1 min after receiving an i.c.v. injection with 1 µg angiotensin II [8] in aCSF, was removed from the experiment.

### Evans blue dye

At the end of the experiment, chicks received an i.c.v. injection of aCSF with 0.1% Evans Blue (ChEBI ID n° 82467), an azo dye with a very high affinity to serum albumin. Because serum albumin cannot cross the blood-brain barrier, the central neural tissue itself will not be stained [9]. Brain samples without Evans Blue apparent in the third ventricle were excluded from analyses.

### Success rate

This protocol has a mortality rate close to zero, and a success rate of 88% (*n* = 224). The success rate is based on the correct and stable placement of the cannula and the drop-out rate during a post-operative period of 14 days.

## Declaration of Competing Interest

The authors declare that they have no known competing financial interests or personal relationships that could have appeared to influence the work reported in this paper.
